# Myeloid Cells Enriched for a Dendritic Cell Population From People Living With HIV Have Altered Gene Expression Not Restored by Antiretroviral Therapy

**DOI:** 10.3389/fimmu.2020.00261

**Published:** 2020-03-04

**Authors:** Shannon M. Murray, Yuwei Zhang, Daniel C. Douek, Rafick P. Sekaly

**Affiliations:** ^1^Human Immunology Section, Vaccine Research Center, National Institute of Allergy and Infectious Diseases, National Institutes of Health, Bethesda, MD, United States; ^2^Vaccine and Gene Therapy Institute Florida, Port St. Lucie, FL, United States

**Keywords:** human immunodeficiency virus (HIV), antiretroviral therapy (ART), pathogenesis, dendritic cells, myeloid cells, monocytes

## Abstract

Antiretroviral therapy (ART) for human immunodeficiency virus (HIV) infections has been designed to optimize CD4 T-cell survival and limit HIV replication. Cell types other than CD4 T cells such as monocytes/macrophage, dendritic cells, and granulocytes (collectively known as myeloid cells), are generally not considered in the development of ART protocols. Myeloid dendritic cells (mDCs) are the most potent inducers of CD4 T-cell activation and central to the regulation of immune responses. mDCs in the blood are decreased in number, altered in function, and implicated in promoting HIV latency in people living with HIV (PLWH). We found that cells enriched for mDC in PLWH had transcriptional changes compared to mDC from HIV uninfected individuals, some of which were not completely restored by ART. In contrast, other mDC functions such as interleukin-1 signaling and type I interferon pathways were restored by ART. Some of the transcriptional changes in mDC not completely reversed by ART were enriched in genes that are classically associated with cells of the monocyte/macrophage lineage, but new single-cell RNA sequencing studies show that they are also expressed by a subset of mDC. A cellular enzyme, acyloxyacyl hydrolase (AOAH), important for lipopolysaccharide (LPS) detoxification, had increased transcription in mDC of PLWH, not restored by ART. It is possible that one reason ART is not completely successful in PLWH is the failure to phenotypically change the mDCs. Thus, inability of ART to be completely effective might involve myeloid cells and the failure to restore mDC function as measured by gene transcription. We suggest that mDC and myeloid cells should be considered in future combination ART development.

## Introduction

Dendritic cells (DCs) and monocytes are innate immune cells of the myeloid lineage that play critical roles in protection against microbial infections (1, 2). DCs internalize microbes in endocytic compartments where microbes are degraded into components. The resulting antigens are presented to T and B cells to stimulate immune responses against that microbe. In contrast, monocytes, which differentiate into macrophage within tissues, are specialized to degrade and scavenge microbes from the host organism (1). Monocytes/macrophage can also present antigens; however, it is important to note that DCs are >100-fold more potent in activating T cells through their antigen-presenting functions than are monocytes/macrophage (1).

DC and monocytes are generated in the bone marrow during hematopoiesis. These two cell types share a common hematopoietic progenitor, the monocyte and DC precursor (MDP) (3–5). Two branches of differentiation are thought to arise from the MDP: one branch to monocytes and another branch to a common DC progenitor (CDP) (6, 7). As we are focusing on DC, it is important to know that the CDP generates at least three DC subpopulations. The three major DC populations are the myeloid DC type 1 (mDC1), 2 (mDC2), and plasmacytoid DC (pDC) (5, 8). mDC2 is the most frequent and is known as conventional DC (6, 7, 9). Recent single-cell RNA sequencing (scRNAseq) of mDC from healthy humans revealed that the mDC2 population is comprised of two transcriptionally distinct subsets, one newly defined, which expressed certain genes traditionally associated with monocyte/macrophage (10). Herein, we concentrate our studies on mDC2, also known as conventional DC type 2 (cDC2) or CD1c^+^ mDC, the largest DC subset. This subset will be called mDC in our studies. We will focus on this subset in the peripheral blood before cells migrate to different tissue types and are designated by some as “precursor-DC” (pre-DC) [reviewed in Collin and Bigley (9)].

A major effect of HIV-1 (herein called HIV) infections is the loss of CD4 T cells, the primary target of direct HIV infection. While HIV does not significantly infect mDC *in vivo* (11), mDCs are altered in function (12) and decreased in number (13–17) in the blood in untreated people living with HIV (PLWH) and simian immunodeficiency virus (SIV)-infected macaques (18, 19). Increased HIV RNA viral loads and disease progression are associated with loss of blood mDC (13–16, 18). There is some indication that mDC may be an important co-factor in the efficient infection of CD4 T cells as *in vitro* studies show they bind virus on their cell surface and are able to transfer virus to CD4 T cells in a mode called “trans” infection (20, 21) [reviewed in Manches et al. (22)].

Antiretroviral therapy (ART) has been developed to limit HIV replication and prevent the loss of CD4 T cells. Unfortunately, ART is not always efficacious as some PLWH fail to reconstitute their CD4 T-cell numbers and become susceptible to opportunistic infections. One component of ART failure may be a result of the incomplete restoration of blood mDC count and function. One can speculate that myeloid cells, and specifically mDC, play a role in HIV persistence. First, plasma levels of two soluble myeloid cell surface molecules, CD14 and CD163, correlate with adverse events, co-morbidities, and disease progression in both ART-treated and treatment-naïve (TN) people living with HIV (PLWH) (23–31) and SIV-infected macaques (32). CD14 and CD163 are shed by myeloid cells (particularly monocytes) after binding to bacterial ligands. It is thought that this myeloid cell surface molecule shedding occurs, in part, because of the elevated levels of the gram-negative bacterial endotoxin, lipopolysaccharide (LPS), in the blood of PLWH. Increased LPS and other bacterial components in the blood of PLWH (33–35) are hypothesized to be a result of increased gastrointestinal (G.I.) tract permeability in PLWH (36) [reviewed in Brenchley and Douek (37, 38)]. Second, generalized T-cell immune activation occurs with chronic HIV infections (39–42) and correlates with HIV disease progression. This immune activation is associated with ART failure, yet its causes remain unexplained. It is possible that mDC, in close contact with T cells, play a role in such immune activation. Thus, due to their close association with T cells, and their changes in PLWH, mDC may be important in sustaining generalized T-cell immune activation that occurs in PLWH. Third, *Mycobacterium tuberculosis* (MTB) is a major opportunistic infection (O.I.) in PLWH. While the incidence of MTB is significantly reduced after ART, by ca. sixty-five percent (43, 44), it is not completely eliminated and still occurs at higher frequencies worldwide in PLWH than in the population at large (43, 44). Studies in mice suggest that mDC are important for immune responses to and clearance of MTB [reviewed in Durai and Murphy (45)], and therefore, their decreased numbers may be a factor in MTB susceptibility PLWH. Thus, mDC loss or alterations may be related to the increased susceptibility to secondary infections in PLWH.

Our work, described herein, was designed to determine whether mDC gene expression is altered in untreated or ART-treated PLWH. While the loss of CD4 T cells has been the dominant explanation for the immune dysfunction that occurs during HIV infections, including susceptibility to O.I.s such as MTB, defects in mDC may also play a role. Some limitations and/or failure of ART might be attributable to changes in mDC and/or myeloid cells.

## Materials and Methods

### Study Participants

All participants included in this study provided written informed consent. This study was approved by either the Institutional Review Boards of the National Institute of Allergy and Infectious Diseases or the Vaccine and Gene Therapy Institute Florida (VGTIFL), as appropriate. In order to obtain a sufficient number of mDC, apheresis was obtained from each donor. Participants were pre-screened for their ability to undergo apheresis. Each participant was required to be in good health and have within, or close to, normal levels of circulating CD4 T cells. Apheresis was performed for all subjects.

### Myeloid Dendritic Cell and Monocyte Purification

mDCs were directly isolated from donors as follows: We identified mDC using the following markers: CD1c^+^, HLA-DR^intermediate to high^, CD11c^high^, CD14^−^, CD19^−^, CD3^−^, CD123^−^, and BDCA 4^−^ (summarized in Supplementary Figure 1). The leukapheresis product was elutriated (Elutra, Gambro) to separate myeloid cells from lymphocytes, according to the manufacturer's protocol (46, 47). The elutriated myeloid cells were then used for the separation of mDC and CD14^+^ monocytes by a magnetic bead-enrichment protocol (DC Enrichment Kit, Miltenyi) and platform (Automacs, Miltenyi) for the isolation of DC. Briefly, the elutriated myeloid cells were incubated with Fc receptor block and BDCA-4 antibody conjugated to magnetic beads for 15 min at 4°C. Cells were washed and then centrifuged at 1,650 rpm for 3 min. The cells were resuspended in wash buffer and depleted of BDCA-4+ plasmacytoid DCs by magnetic bead separation. The remaining cells were washed and incubated with a magnetic bead-conjugated CD19 antibody and a biotin-conjugated CD1c antibody for 15 min at 4°C. Cells were then washed and centrifuged for 3 min at 1,650 rpm and resuspended in wash buffer (PBS with 2% BSA and 0.09% azide). The cells were depleted of CD19^+^ B cells. The remaining cells were centrifuged and incubated with an anti-biotin antibody to capture CD1c^+^ cells. In some cases, when elutriation did not result in cell fractions that were highly enriched for monocytic cells, as determined by an automated cell counter (Countess, Invitrogen), cells were further separated by Ficoll and then enriched by antibody-conjugated bead separation followed by FACS sorting with the same procedures as described for the elutriated-only product. This was performed for two samples. For all samples, monocytes were isolated from the cell fraction remaining after magnetic bead-conjugated antibody depletion of CD1c^+^ cells, B cells, and plasmacytoid DC, as per the manufacturer's protocol (Miltenyi).

It is known that using bead enrichment alone yields DC preparations that are contaminated with other cell types (48), and for this reason, we used a subsequent FACS purification step. The magnetic bead enriched mDCs or monocyte populations were further purified by fluorescence-activated cell sorting (FACS; Becton Dickinson, FACSARIA). Viable mDC, as measured by the viability dye, aqua (Invitrogen), were sorted on the basis of size (side scatter and forward scatter) and the following markers (obtained from BD Biosciences): CD11c^high^, HLA-DR^intermediate to high^, CD14^−^, CD123^−^, CD3^−^, CD56–, and CD19^−^. Monocytes were sorted on the basis of size (side scatter and forward scatter), viability (aqua vs. side scatter), and the following markers: HLA-DR ^intermediate to high^, CD14^hi^, CD3^−^, CD56^−^, CD123^−^, and CD19^−^ (Supplementary Figure 1). We showed that monocytes were confirmed to be ≥97% pure by post-sort analysis and estimate the mDC had the same level of purity, although there were not sufficient mDCs to analyze by post-sort analysis. Cells were sorted directly into RLT buffer (guanidinium thiocynate) with 1% β-mercaptoethanol (Qiagen) and stored at −80°C until RNA isolation. All cells were sorted as replicates, in at least duplicates.

### mRNA Extraction and cRNA Generation

RNA was extracted from RLT Buffer (Qiagen)-treated mDC using the RNeasy Micro kit (Qiagen), according to the manufacturer's protocol. Total RNA was checked for quantity and quality using a NanoDrop 2000c spectrophotometer (Thermo Fisher Scientific) and Experion automated electrophoresis system (Bio-Rad Laboratories). Samples that were not of sufficient quality and/or quantity were not further studied. mRNA from each sample was amplified using the MessageAmp II aRNA Amplification kit (Ambion) according to the manufacturer's protocol. This involved oligo DT primers and optimized MMLV RT, T7 RNA polymerase, and biotinylated nucleotides.

### Microarray Analyses

Subsequently, 750 ng of the biotinylated amplified cRNA was hybridized to the HumanRef-8 v3.0 or Human Transcriptome (HT)12_V4_Beadchip Microarrays (Illumina) at 58°C for 20 h and then quantified using the iScan System (Illumina).

### Statistical Analyses

The statistical analysis of the microarray data was performed essentially as described (49). Analysis of the Genome Studio output data was conducted using the R statistical language (R Development Core Team) and software packages from Bioconductor (50). First, arrays displaying unusually low median intensity or low correlation relative to the bulk of the arrays were discarded from the rest of the analysis. Quantile normalization was applied followed by a log_2_ transformation. Some samples were acquired at different times, using different versions of the HT gene arrays. For this reason, batch correction was employed to normalize for interarray variability. Batch effect subtraction was performed using the ComBat procedure (51). The LIMMA package (Bioconductor) (52) was used to fit a linear model to each probe and to perform a (moderated) Student's *t* test on various differences of interest.

Ingenuity pathways analysis (IPA, Qiagen) and gene set enrichment (GSEA) were used to identify pathways altered in the samples being compared. Two-dimensional scaling analysis was done in Bioconductor. The expected proportions of false positives, the false discovery rates (FDR), were estimated from the unadjusted *p*-value using the Benjamini and Hochberg method (53). The microarray data are available through the National Center for Biotechnology Information Gene Expression Omnibus (GEO) under accession no. GSE139559.

### Quantification of Intracellular IL-1α and Phosphorylated p38 and IκKγ

Peripheral blood mononuclear cells (PBMC) from study subjects were isolated by density gradient centrifugation (Histopaque, GE Healthcare) of aphereses obtained from TN HIV-infected or HIV-uninfected individuals. PBMC were stained with the following cell surface molecules: HLA-DR APC-Cy7 (BD Biosciences), CD11c APC (BD), CD14 Pacific Blue (BD), CD3 PerCP-Cy5.5 (BD), and CD19 ECD (Beckman Coulter) for 30 min and washed twice in FACS buffer (2% fetal bovine serum in PBS). The samples were then resuspended in complete media (RPMI 1640) and stimulated with interleukin-1 (IL-1; 10 ng/ml; R&D Systems) for 15 min at 37°C. Following the incubation, the samples were centrifuged at 1,800 rpm for 5 min at RT and then fixed with pre-warmed BD Cytofix buffer (BD Biosciences) for 10 min at 37°C. The fixed samples were then permeabilized in cold BD Phosphoflow™ Perm Buffer III (BD Biosciences). Samples were washed in FACS buffer and then stained with BD Phosphoflow^TM^ phosphorylation intracellular p38-P and IκKγ-P antibodies (BD Biosciences). After two additional washes with FACS buffer, the samples were analyzed by flow cytometry using an LSR II (BD) and DIVA software. mDCs were identified by expression of the following markers: HLA-DR^intermediate to high^, CD11c^high^, CD14^−^, CD3^−^, CD19^−^, CD56^−^, and CD123^−^. Analyses were performed using Flow Jo version 10 (Tree Star) to assess IL-1α levels, and p38 and IκKγ phosphorylation levels after flow cytometry gating on mDC populations.

Intracellular IL-1α expression was determined by intracellular staining with a PE-conjugated antibody to IL-1α (BD Pharmingen). PBMCs were stimulated with CL097 (1 μg/ml; a TLR 8 ssRNA analog; Invivogen) or LPS (100 ng/ml; Sigma-Aldrich) for 16 h. Cells were stained with antibodies to the aforementioned cell surface molecules for 30 min and washed twice in FACS wash buffer, after centrifugation at 1,800 rpm for 5 min followed by a fixation in 2% PFA in PBS. Then cells were permeabilized by a wash with 0.05% saponin in PBS followed by centrifugation at 1,800 rpm for 5 min. Cells were then stained with IL-1α antibody in PBS with 0.05% saponin for 30 min at room temperature. Cells were then washed twice in FACS wash buffer and then fixed in 2% PFA in PBS. Sample data were collected as described above.

## Results

### Isolation of mDC From PLWH and HIV-Uninfected Study Participants

The participants are a cross section of HIV-infected persons who were seen at the Vaccine Research Center Clinic, NIAID, NIH (Bethesda, MD) and who were infected with HIV for times ranging from an estimated 1 to 20 years and who met the criteria of having within, or close to, normal levels of CD4 T cells in the blood, i.e., ~500–1,500 CD4 T cells/μl (Table 1). Three of the six HIV-infected persons were untreated, while the others received ART regimens for times ranging between 2 and 13 years. The estimated time since HIV infection was between 1 and 20 years for the TN participants and between 2 and 15 years for the ART-treated participants. All ART-treated PLWH had been treated at least 2 years before enrolling in this study. Two of the ART-treated participants were virally suppressed, <50 plasma viral RNA copies/ml, and one ART-treated individual had a plasma viral RNA load of ~400 copies/ml, which is considered a relatively low viral load. Since only one time point was examined, it is not known whether the ca. 400 copies/ml represents an ongoing low level of viral replication or a “viral blip.” Viral blips are known to occur in ~20% of ART-treated PLWH (54).

**Table 1:** Study participants for mDC isolations.

**HIV status**	**Age (years)**	**Gender**	**Treatment**	**Estimated time since HIV± (years)**	**Time since ART (years)**	**Viral RNA copies/ml**	**CD4 T cells/μl**
HIVp1	28	M	ART	2	2	<50	447
HIVp2	52	M	ART	15	13	392	654
**HIVp3**	51	F	**TN**	**12**	**NA**	**5,970**	**641**
HIVp4	41	M	ART	12	6	<50	619
**HIVp5**	21	M	**TN**	**1**	**NA**	**304, 742**	**513**
**HIVp6**	52	M	**TN**	**20**	**NA**	**19,998**	**430**
HIVn1, 2, 3, 4	§	§	NA	NA	NA	NA	*

The cohort included antiretroviral therapy (ART)-treated and treatment-naïve (TN) (in bold) people living with HIV (PLWH) as well as HIV-uninfected persons. HIVp, HIV positive; HIVn, HIV negative; NA, not applicable; §, unavailable; *within normal ranges for CD4 T cells.

Of the untreated HIV-infected participants, plasma viral RNA loads were of a wide range, from 6,000 to 300,000 copies/ml (Table 1). It is generally accepted that long-term non-progressors (LTNPs) are PLWH who have been HIV infected for ≥7 years and maintain CD4 T cells ≥500 cells/μl of blood [reviewed in Gurdasani et al. (55)]. Subject HIVp3 had been infected for an estimated 12 years and had CD4 T-cell levels of ca. 640 cells/μl and, thus, is considered an LTNP. Participant HIVp6 was HIV infected for over 20 years and had CD4 T cells of 430 cells/μl and, thus, is not considered an LTNP. The third untreated HIV-infected participant, HIVp5, was infected for only 1 year. In addition to the HIV-infected participants, four HIV uninfected healthy subjects, with CD4 T-cell levels within the normal range, whose blood was available through the NIH Clinical Center, were also studied as controls (Table 1). All individuals were subjected to apheresis.

As mDCs are sensitive to cryopreservation, we obtained mRNAs from freshly isolated mDCs as well as from monocytes, as described (see Materials and Methods section). We identified mDC using the following markers: CD1c^+^, HLA-DR^intermediate to high^, CD11c^high^, CD14^−^, CD19^−^, CD123^−^, CD56^−^, and BDCA 4^−^ (Table 2 and Supplementary Figure 1). Monocytes were identified by the following markers: CD1c^−^, HLA-DR^intermediate to high^, CD14^high^, CD19^−^, CD123^−^, CD56–, and BDCA-4^−^ (Supplementary Figure 1). We isolated mRNA from these purified cells, generated cRNA libraries, and performed gene expression array analyses.

**Table 2:** Strategy for the isolation of mDC from PLWH and HIV uninfected persons.

**Cell surface marker**	**Enrichment**	**FACS**
CD1c^+^	x	
BDCA-4^−^	x	
CD19^−^	x	
HLA-DR^intermediate−high^		x
CD11c^+^		x
CD14^−^		x
Lin (CD3^−^, CD56^−^, CD19^−^, CD123^−^)		x

### Altered Gene Expression in mDC From Untreated and ART-treated PLWH

**Figure 1:**
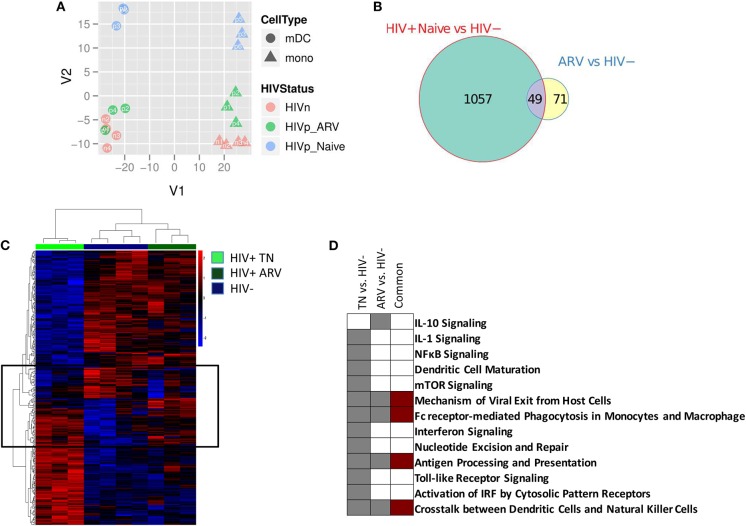
Myeloid dendritic cells (mDCs) from treatment-naïve (TN) and antiretroviral (ARV)-treated people living with HIV (PLWH) have a different gene transcription compared to mDCs from HIV-uninfected individuals. **(A)** Principal component analysis of the gene expression profiles for the top-most variable genes between mDCs and monocytes from TN PLWH, ARV-treated PLWH, and HIV-uninfected individuals. **(B)** The Venn diagram indicates the number of common and unique genes (|FC| > 2 and *p* < 0.05) between the mDC contrasts from PLWH and HIV-uninfected persons. **(C)** Heatmaps of differentially expressed genes of mDC from treatment-naive (TN) or ARV-treated PLWH. Differentially expressed genes with |FC| > 2 and *p* < 0.05 are shown. Highlighted on the heatmap, indicated by a box, are those genes commonly altered in mDC from TN and ARV-treated PLWH compared to HIV-uninfected persons. **(D)** Pathway alterations in mDC from TN and ARV-treated PLWH compared to mDC from HIV uninfected persons. Ingenuity pathway analysis (IPA), as described in the Materials and Methods section, was used to identify those pathways enriched in the differentially expressed genes in each contrast. The complete list of pathways associated with these contrasts is provided in the Supplementary Materials, Supplementary Figure 2. ARV, antiretroviral therapy; HIVn, HIV negative; HIVp_Naive, HIV positive, treatment naïve; HIVp_ARV, HIV positive, antiretroviral-treated.

We compared the gene expression profiles of mDC and monocytes from treatment-naïve (TN) PLWH, ART-treated PLWH, and those from HIV-uninfected individuals. The principal component analysis (PCA) describes the variation in gene expression between samples of interest. Using the PCA, we found that cell type-specific gene expression represented the greatest variation between the samples. This is graphically represented in PC1 (or the x axis) in Figure 1A, with mDCs and monocytes separating from each other, for all individuals (Figure 1A). Other gene expression variations between samples are accounted for in PC2 (or the y axis). Samples grouped together based on HIV treatment status in PC2. mDCs and monocytes from TN PLWH grouped together in PC2, as did mDCs and monocytes from ART-treated PLWH, and as did those from HIV-uninfected persons (Figure 1A). However, mDCs and monocytes from TN individuals had the greatest divergence from all of the other groups. For example, mDCs and monocytes from ART-treated individuals clustered more closely to those from HIV-uninfected individuals than those from TN PLWH. The transcriptional profile for mDCs and monocytes from ART-treated HIV-infected individuals, however, segregated distinctly from HIV-uninfected individuals (Figure 1A).

We performed a differential gene expression (DEG) analysis between mDC from TN or ART-treated PLWH to those from HIV-uninfected persons (using a supervised analysis). We found that 1,106 genes were differentially expressed between mDCs from TN PLWH compared to those of HIV-uninfected individuals (FC, fold change ≥2, *p* ≤ 0.05; Figure 1B). We also found that 120 genes were differentially expressed in mDCs from ART-treated HIV PLWH compared to those of HIV-uninfected individuals (Figure 1B). Of the 120 genes altered in mDCs from ART-treated PLWH compared to those of HIV-uninfected individuals, 71 of these were unique to ART treatment, while 49 of these were shared with mDCs from TN PLWH (Figure 1B).

### mDC Biological Pathways Are Altered in Untreated and ART-Treated PLWH

When evaluating the differentially expressed genes in the mDCs from TN PLWH compared to those from HIV-uninfected persons, we found that the mDCs from ART-treated PLWH grouped more closely to the mDCs from HIV-uninfected individuals (Figure 1C). However, mDCs from ART PLWH formed a distinct group or branching from HIV-uninfected individuals, as depicted in the heatmap shown (Figure 1C). The ART profile was more akin to the HIV-uninfected profile, but there are groups of genes that differed from those of HIV-uninfected individuals, many of which were in common with those of TN HIV-infected persons (Figure 1C). This was also the case for the CD14^+^ monocytes (data not shown). We then determined whether the genes that changed in the three groups were included in specific biological pathways. We used the ingenuity pathways analysis (IPA) database to identify such pathways. The most highly represented pathway alteration in mDCs in TN PLWH relative to HIV-uninfected persons was the interferon pathway (Figure 1D and Supplementary Figure 2). We also found significant alterations in other pathways such as the viral nucleotide excision repair and mechanisms of viral exit from host cell pathways. There were also alterations in the antigen processing and presentation and mTOR pathways as well as in the IL-1 signaling pathway (summarized in Figure 1D and Supplementary Figure 2).

In mDCs from ART-treated PLWH, the IL-4 and IL-10 pathways were altered relative to HIV-uninfected persons, and these were not significantly altered in TN PLWH (Figure 1D and Supplementary Figure 2). Pathway alterations shared between mDCs from ART-treated and TN PLWH included the FC receptor-mediated endocytosis pathway and antigen processing and presentation (Figure 1D). Thus, while the majority of pathway changes in mDC appeared to be reversed by ART, there were some pathways that were only found in the mDCs from ART-PLWH and some pathways that were shared between the mDCs from ART and TN PLWH relative to HIV-uninfected individuals (Figure 1D and Supplementary Figure 2).

### A Type I IFN Transcriptional Signature in mDCs From TN PLWH Is Mostly Reversed by ART

**Figure 2:**
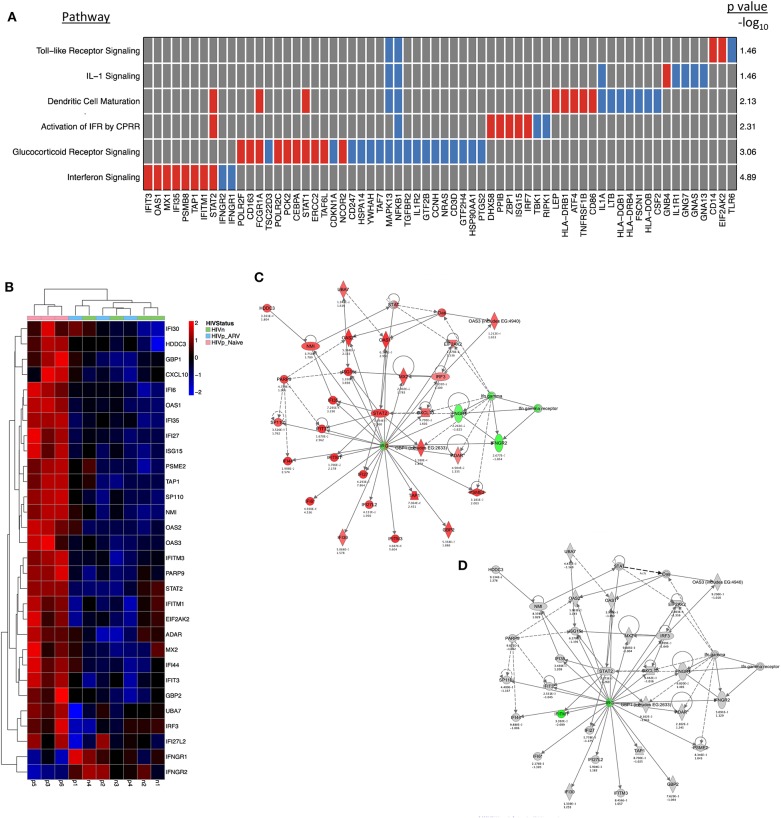
Interferon signaling is the most significantly altered pathway in mDCs from untreated PLWH compared to HIV-uninfected individuals and is mostly restored to HIV-uninfected levels in mDCs from ARV-treated PLWH. mDCs from treatment-naïve (TN) PLWH have type I IFN pathway transcriptional signatures. **(A)** A pathway heatmap showing pathways altered, including the IFN pathway, the most significantly altered pathway, in mDCs from TN PLWH compared to HIV-uninfected persons. **(B)** Heatmap showing interferon-response genes in the ingenuity pathway analysis (IPA) network for antimicrobial and inflammatory response for mDCs from TN and ARV-treated PLWH and HIV-uninfected individuals. **(C,D)** The same IPA genes in their networks, showing those gene alterations in **(C)** mDCs from TN PLWH and **(D)** ARV-treated PLWH relative to HIV-uninfected persons. In ARV-treated PLWH, type I IFN gene transcription is mostly restored to HIV-uninfected levels; however, some **(D)** type I IFN genes are reversed in expression and downregulated relative to HIV-uninfected levels. Numbers below each gene node indicate the *p* value and below that (log_2_) fold change of that gene's expression relative to HIV-uninfected levels.

We observed that mDCs from TN HIV-infected individuals had profound transcriptional upregulation of a type I interferon (IFN) response (Figures 2A–C). When we scrutinized networks of IFN-inducible genes of mDCs from TN PLWH, we found a significant induction of many of the IFN-inducible genes (Figures 2A–C) including several that are components of the innate cell intrinsic antiviral machinery (MX1, OAS1, IFIT3). STAT1 and STAT2, which activate type I IFN transcriptional networks, with STAT1 also involved in type II IFN responses, were uniformly upregulated in mDC from TN PLWH (Figures 2A–C). Surprisingly, other components of the IFNγ (type II IFN) pathway such as IFN gamma receptor 1 (IFNγR1) and 2 (IFNγR2) were slightly downmodulated in mDCs from TN HIV-infected subjects (Figures 2A,C).

The type I IFN pathway in the mDCs from ART-treated PLWH was similar to the mDCs from HIV-uninfected persons. However, the mDCs from ART-treated PLWH did not exhibit complete restoration of the type I IFN pathway to expression levels observed in HIV-uninfected individuals (Figures 2B,D). Three genes (IFITM1, IRF7, and IRF9), which are involved in regulation and transcriptional activation of interferon responses, were all reversed in expression change, and were downregulated relative to that of mDC from HIV-uninfected individuals (Figures 2B,D).

### IL-1 Signaling Is Suppressed in mDCs From TN PLWH

**Figure 3:**
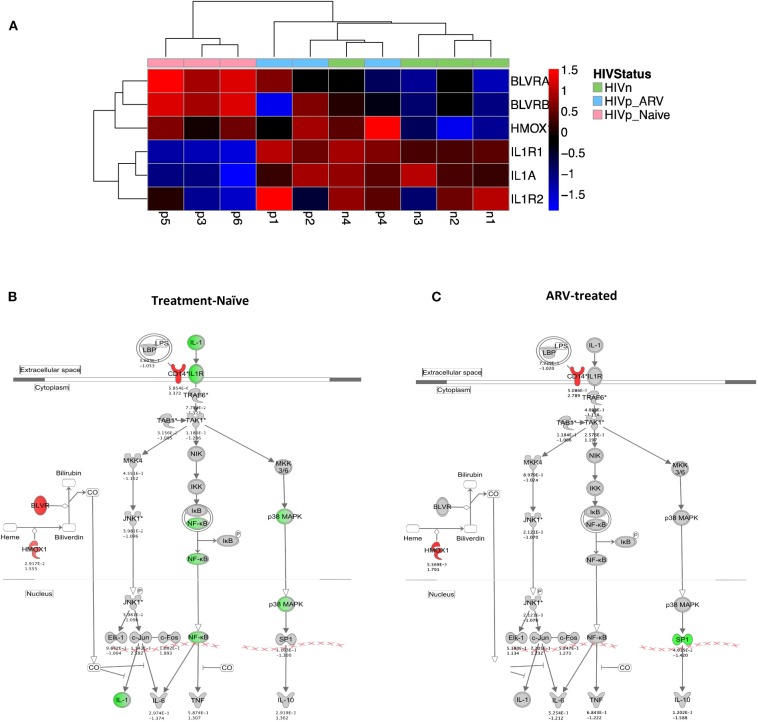
The IL-1 signaling pathway is transcriptionally suppressed in mDC from treatment-naïve PLWH. **(A)** Heatmap and **(B)** network illustrating key IL-1 pathway genes and their modulators. **(A,B)** IL-1α and its receptors are transcriptionally suppressed in mDCs from TN PLWH compared to ARV-treated or HIV-uninfected persons. **(B,C)** Negative regulators of IL-1 signaling, including hemoxygenase (HMOX-1) and CD14, components of the IL-10 pathway, are **(B)** upregulated in mDCs from TN PLWH. **(C)** In mDCs from ARV-treated PLWH, levels of suppressors of IL-1 signaling are not completely restored to levels of mDCs from HIV-uninfected persons. **(B,C)** Shown is the ingenuity pathway analysis (IPA) pathway, showing both IL-1- and IL-10-associated genes. Numbers below each gene node indicate *p* value and below that (log_2_) fold change of that gene's expression relative to HIV-uninfected levels.

**Figure 4:**
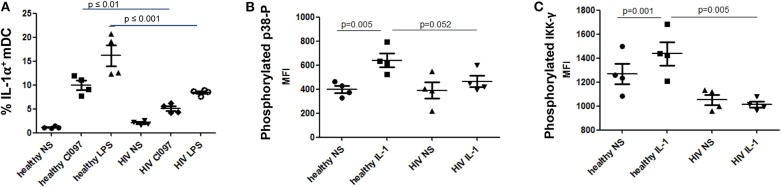
Diminished interleukin-1 (IL-1α) production and suppressed NF-κB pathways in mDCs from treatment-naïve PLWH after IL-1α or microbial stimulation. Impaired **(A)** IL-1α production following microbial stimulation of mDCs from untreated PLWH. **(B)** IL-1-induced signaling in mDCs from untreated PLWH show decreased NF-κB activation as indicated by **(B)** p38 phosphorylation and **(C)** IKK**γ** phosphorylation. Phosphorylated p38 and IKKγ were measured by flow cytometry, and the mean fluorescence intensity (MFI) of the specific antibodies are reported for mDC, as described in the Methods and Materials section. CL097, a TLR 7/8 agonist. IL-1 refers to IL-1α-stimulated cells, as described in the Materials and Methods section. NS, no stimulation; LPS, lipopolysaccharide.

We identified transcriptional downmodulation of the IL-1 pathway in TN HIV-infected individuals (Figures 3A–C). We found several genes that encode cytokine receptors belonging to the interleukin 1 receptor family, including IL1RA, and IL1R2 as well as IL1A, itself, were downregulated in mDCs from TN PLWH compared to HIV-uninfected individuals (Figures 3A,B). The IL-1 signaling pathway plays a critical role in response to microbial infection [reviewed in Mayer-Barber and Yan (56)]. In order to validate some of the transcriptional changes seen, we looked at IL-1α protein levels in activated mDC TN PLWH (Figures 4A–C). We sought to determine whether mDCs from TN HIV-infected individuals were impaired in IL-1α production after microbial stimulation or in IL-1 signaling pathways downstream of IL-1α stimulation. In order to address this, we first tested mDCs obtained from TN PLWH or HIV-uninfected persons, before or after microbial exposure *in vitro*. We measured intracellular IL-1α production in mDCs from HIV-uninfected individuals and compared this to that of TN HIV-infected individuals. We found no significant difference in basal levels of IL-1α protein expression (Figure 4A). However, upon stimulation with a single-stranded RNA toll-like receptor (TLR) 7/8 agonist, CL097 (*p* < 0.01), or TLR4 (and CD14) agonist LPS (*p* < 0.001), we found a statistically significant decline in IL-1α production among HIV-infected TN individuals compared to that of HIV-uninfected individuals (Figure 4A).

IL-1α stimulation activates the NF-κB pathway, which is essential for innate immune cell function [reviewed in Caamano and Hunter (57) and Dorrington and Fraser (58)]. Activation of NF-κB requires phosphorylation of p38 (MAPK) and IκK. Thus, we quantified levels of phosphorylated p38 and IκKγ after IL-1α stimulation of mDC, to determine whether activation of the NF-κB pathway by IL-1α stimulation was impaired in mDCs from TN PLWH as we had observed in gene transcription. Following stimulation of mDCs from HIV-infected TN individuals with IL-1α, we also observed a statistically significant suppression of p38 MAPK activation and NF-κB activation by IκKγ phosphorylation between HIV-uninfected and TN HIV-infected individuals (*p* = 0.052 and *p* = 0.005, respectively) (Figures 4B,C). Similarly, one previous study demonstrated suppressed IκKγ phosphorylation after TLR ligand stimulation in DCs from healthy individuals in an HIV plasma-transfer model (12). We found that mDCs from chronic TN HIV infections have suppressed IL-1α activation in response to microbial stimulation and an impaired IL-1 pathway in response to IL-1α stimulation (Figures 4A–C).

### IL-1 Signaling Pathway Is Mostly Restored in mDCs From ART-Treated PLWH

Three known endogenous suppressors of the IL-1 pathway, Biliverdin A and B (BLVRA and BLVRB) and hemoxygenase-1 (HMOX-1) are associated with the IL-10 pathway. All three were upregulated in TN and ART-treated PLWH compared to HIV-uninfected individuals (Figures 3A,C). Unlike the IL-1 receptor family genes in mDCs from ART-treated individuals that were like (grouped with) HIV-uninfected individuals in expression patterns (Figure 3A), the BLVR genes, and most significantly, the HMOX-1 gene expression, were more akin to mDCs from TN PLWH than from HIV-uninfected persons (Figures 3A,C). Therefore, ART is mostly reversing the IL-1 pathway gene transcription to HIV-uninfected levels, but not all of the suppressors of the IL-1 pathway, in mDCs.

### mDC Gene Transcription Including CD14 Is Not Restored by ART in PLWH and Indicates Altered Myeloid Cell Differentiation Pathways

**Figure 5:**
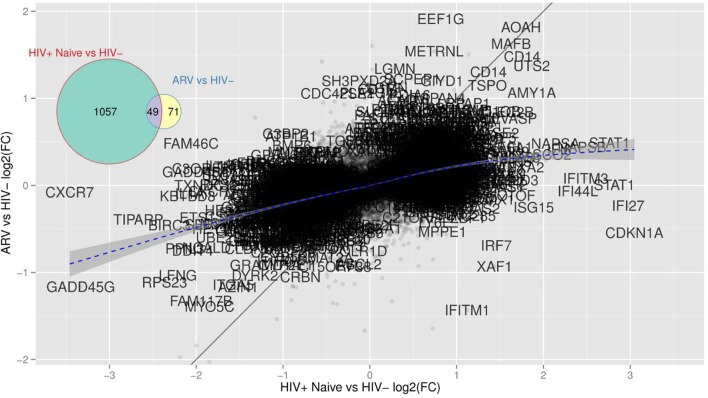
mDCs from TN PLWH have transcriptional alterations not restored in mDCs from ARV-treated PLWH to HIV-uninfected levels. This scatterplot compares mDC gene expression from TN or ARV-treated PLWH relative to HIV-uninfected persons expressed as (log_2_) fold changes. There are far more genes with high fold changes in the TN than in the ARV group, indicating that mDCs from ARV-treated PLWH is closer to the HIV-uninfected group. There are genes “maintained,” i.e., HIV-elicited genes that are unaffected by the therapy (AOAH, MAFB, CD14, all those close to the identity line or “common” genes) and genes whose differential expression is strongly suppressed by the therapy (all those close to the x-axis and, therefore, unique to TN PLWH), and those that are an effect of the therapy alone (close to the y-axis and unique to ARV). A gene like IFITM1 is reversed by therapy as it lies close to the y = –x line.

Similar to HMOX-1, a gene that was altered in mDCs from both TN and ART PLWH relative to HIV-uninfected individuals, we found that there were other genes commonly altered in HIV infection relative to HIV-uninfected persons. We examined those genes commonly altered in both TN and ART-treated PLWH. The multidimensional scaling plot (MDS) (Figure 5) shows those genes commonly altered in mDCs from untreated and ART-treated PLWH as well as those not restored to HIV-uninfected levels with ART treatment. Strikingly, there exists a group of genes that are commonly upregulated and relatively unaffected in expression levels in TN and ART-treated PLWH. Some of the genes included CD14, acyloxyacyl hydrolase (AOAH), and MAFB (V-maf musculoaponeurotic fibrosarcoma oncogene homolog B). A gene such as IFITM1 is upregulated in mDCs from TN PLWH but downregulated in ART-treated PLWH compared to HIV-uninfected individuals (Figure 5). The complete set of 49 genes not restored by ART is shown (Table 3).

**Table 3:** List of genes whose transcription is commonly altered in mDCs from TN and ART-treated PLWH relative to HIV-uninfected persons.

AAMP	POLR1D
CECR5	FAM109A
STAB1	BECN1
LRPAP1	SEPT3
GTPBP8	COASY
CYP2E1	NT5DC3
SLC2A9	SFRS5
HK3	MAFB
MFSD7	CENTD2
ZNF362	AFF3
DGCR8	NCALD
EMILIN2	TBC1D9B
SFRS6	CKAP4
HLA-DRB5	MGAT4B
HSPC111	C1QB
AOAH	RIOK1
HLA-DRB1	MYO5C
PRPF31	TOMM7
SYTL3	ARMCX1
CD163	TSPO
FAM117B	GIYD1
CD14	FAM89A
GRK6	TSPAN4
ARS2	ATP6V0B
WAC	

**Figure 6:**
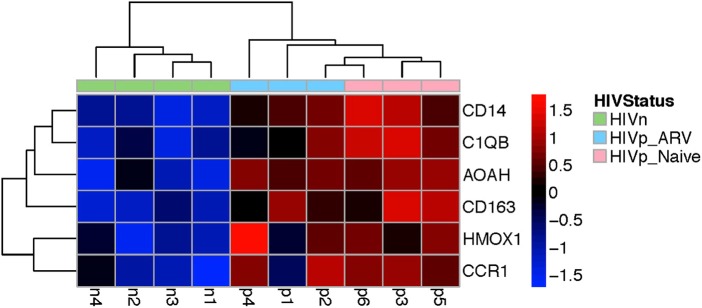
Genes commonly upregulated in mDCs from TN and ARV-treated PLWH included those classically associated with the monocyte/macrophage lineage, myeloid cell differentiation, and inflammatory response. This heatmap shows a subset of those genes commonly upregulated in mDCs from TN or ARV-treated PLWH relative to HIV-uninfected persons. Supplementary Figure 3 illustrates, in more detail, the annotated functions of the genes shown.

The genes were commonly altered in mDCs from ART-treated and TN HIV-infected persons mapped to myeloid cell lineage and differentiation (Supplementary Figure 3), using GSEA analysis, which annotates genes according to modules (datasets annotated by function and coordinated expression). The modules commonly altered in mDC from TN and ART-treated PLWH were (1) myeloid lineage and (2) inflammation I and (3) inflammation II (Supplementary Figure 3). Prominently, some of those genes coordinately upregulated are those most classically associated with a monocyte/macrophage lineage including CD14, CD163, C1QB, and MAFB, and inflammatory response, such as AOAH (Figure 6 and Supplementary Figure 3), also associated with LPS response, as further discussed in the Discussion section. Overall, these results indicate that ART does not completely return transcription in mDCs from ART PLWH to levels seen in HIV-uninfected people.

### Summary

Our data analyzing mDC gene expression indicated that two major pathways were mostly, but not completely, reversed by ART. These pathways are the IL-1 and type I IFN pathways. Surprisingly, some components of the type I IFN pathway were transcriptionally downregulated in mDCs from ART PLWH compared to HIV-uninfected persons. There were transcriptional changes common to mDCs of ART-treated and TN PLWH. Those genes mapped to myeloid cell differentiation and inflammation pathways and included AOAH, and to genes classically associated with a monocyte/macrophage lineage and included CD14, C1QB, CD163, and MAFB. Since recent data from others indicate that there is a subset of mDCs that express these RNA transcripts classically associated with monocyte/macrophage, further studies will need to be done to determine whether the changes we observe are due to alterations in monocytes/macrophages in PLWH that render them more phenotypical like mDCs or expansion of the newly delineated mDC “inflammatory” subpopulation in PLWH. Since our populations of mDC might include monocytic/macrophage precursors, future studies on more purified mDCs remain to be done to determine whether these are monocytes/macrophages or a subpopulation of mDCs. In either case, we found that ART does not restore myeloid cell transcription to that of HIV-uninfected persons.

## Discussion

This work, indicating changes in mDC associated with HIV infections, need to be considered in the development of ART. In this report, we compared gene expression in mDCs from untreated and ART-treated PLWH, who were chronically HIV infected (≥1 year post infection), compared to HIV-uninfected persons. Surprisingly, we found differences in gene expression in mDCs from ART-treated as well as untreated PLWH compared to that of uninfected healthy persons. This indicates that ART does not completely restore transcriptional changes that occur with HIV infections. The data presented herein reveal those 49 genes whose altered expression is common to ART and untreated HIV infections. Some of these genes are primarily considered to be indicative of a classical monocyte/macrophage lineage rather than a DC lineage. These genes include CD14, CD163, and the transcription factor, MAFB. We isolated mDCs by the canonical markers used to delineate DC at the time of the study. Our method employed an antibody-conjugated bead enrichment for CD1c^+^ myeloid cells followed by FACS for mDCs based on size, CD11c^+^, HLA-DR^intermediate−high^, and lineage negative (CD3^−^, CD19^−^, CD56^−^) as well as CD14^−^ markers.

One explanation for our finding is related to a recent study of single-cell RNA sequencing (scRNAseq) of DC (10). This study showed that the CD1c^+^ mDC that we studied here is made up of two transcriptionally distinct mDC subpopulations in healthy individuals (10). The previously unidentified minor mDC subpopulation called “CD1c^+^_B,” or “inflammatory cDC2” expresses monocyte/macrophage genes including CD14 RNA in the absence of CD14 protein on the cell surface, which is what we found in our study. Villani et al. also found higher levels of CD163 and HMOX-1 in this mDC subpopulation than in classical CD1c^+^ mDC [reviewed in Collin and Bigley (9)], similar to genes we observed whose transcription was increased in mDCs from PLWH. Thus, it is possible that the mDCs from PLWH have an increase in this “inflammatory” mDC subpopulation. Another report showed a CD1c^+^ mDC subpopulation that expressed higher levels of MAFB, a transcription factor that had previously been ascribed to monocyte lineage that we also observed with increased transcription in ART-treated and TN PLWH (9, 59). This leads to the possibility that this newly identified mDC population that expresses genes previously associated with monocyte/macrophage, and whose role in health and disease has not yet been established, is an mDC subpopulation expanded in ART-treated and TN HIV infections.

Of those 49 genes altered in mDC from untreated and ART-treated PLWH, we observed some of the highest increases in transcription of the acyloxyacyl hydrolase (AOAH) gene (fold change = ~4). AOAH is a host lipase, which deacylates the gram-negative bacterial outer membrane component, LPS, rendering it less immunostimulatory to the host [reviewed in Munford et al. (60)]. AOAH is primarily expressed by antigen-presenting and phagocytic cells, including mDCs and monocytes (60, 61). AOAH mRNA is increased with LPS exposure of macrophage *in vitro* and *in vivo* (59). AOAH prevents prolonged responses to LPS when overexpressed in mouse DC (62, 63) and protects mice from inflammation-induced injury during gram-negative bacterial infections (59) as well as from gram-negative bacterial infection (63). Thus, the role of AOAH in HIV infections is somewhat paradoxical as PLWH are more susceptible to secondary infections, yet AOAH is associated with protection from gram-negative bacterial infections, at least in murine studies. Research has found a short nucleotide polymorphism (SNP) in an intron of the AOAH gene to be correlated with HIV RNA levels in PLWH (64). It may be that AOAH causes changes in mDC or myeloid cells that alters their ability to stimulate antiviral immunity, but this will need to be addressed in future studies targeted at understanding the role of AOAH in PLWH.

Some systems indicate that type I IFNs suppress IL-1 pathways, leading to increased susceptibility to bacterial and fungal infections (65–67). A type I IFN transcriptional profile in blood cells occurs in active MTB infection and is associated with MTB disease severity (68). The increased type I IFN transcriptional profile, alongside a suppressed IL-1 signaling pathway that we observed in mDC from TN PLWH, could be involved in enhancement of O.I.s such as MTB infections in PLWH. In this study, we found that ART restored type I IFN and IL-1 signaling pathways to near-HIV-uninfected levels. However, we found ~70 genes altered in myeloid cells enriched for mDC in ART-treated PLWH compared to HIV-uninfected persons that were not in common with mDCs from untreated PLWH. Given the use of widespread use of pre-exposure prophylaxis (PrEP), these ART-specific changes should be further investigated for the effects of ART alone on myeloid cells.

The CD14 cell surface protein in HIV-uninfected persons has been used as a marker to differentiate blood monocytes and mDCs since monocytes have CD14 on their cell surface, whereas mDC generally do not. Here, we show a myeloid cell population that expresses CD14 RNA in the absence of significant levels of CD14 protein on the cell surface in PLWH. There are at least two ways to explain these results other than expansion of the newly described CD14 RNA-expressing mDC subpopulation in PLWH, as described above. CD14 is either shed from cells or internalized after binding to LPS (69, 70). This calls into question the utility of using the CD14 antibody as a marker to exclude, identify, or differentiate myeloid cell types in PLWH. Alternatively, a CD14^low^ population of monocytes, known to be expanded in both ART-treated and untreated PLWH, albeit to a lesser extent after ART (71–73), may have been included in our mDC population in PLWH. Inclusion of an additional cell surface marker, CD16, will help exclude CD14^low^ monocytes from the mDC population as CD14^low^ monocytes co-express CD16 monocytes (74). For these reasons, we will refer to the myeloid cell population we have isolated as myeloid cells enriched for mDC. Importantly, we found common transcriptional changes in an enriched myeloid cell population in both ART and TN PLWH compared to HIV-uninfected individuals. Given more recent findings of two classes of mDC, it should be possible in the future to determine whether this newly defined mDC subpopulation is expanded in untreated and ART-treated PLWH, or whether CD14 surface protein is internalized or shed from CD14 RNA-expressing cells that bind LPS in PLWH.

We found that ART does not restore gene transcription in mDCs from PLWH to HIV-uninfected levels. It may be that this represents changes in myeloid cell subpopulations that occur in TN and ART-treated PLWH. Even so, these transcriptional changes are not restored to HIV-uninfected levels with ART. It is known that high levels of CD14 and CD163 in the blood correlate with poor outcomes in including cardiovascular adverse events, fibrosis, and neurological abnormalities in ART-treated as well as TN PLWH [reviewed in Anzinger et al. (29)]. The mDC population that we have identified here may be responsible for the heightened levels of these proteins observed in PLWH, and thus targeting this myeloid cell population in ART would be important. Preliminary data from our lab indicate that plasma LPS levels correlate with CD14 RNA levels in the enriched mDC population in PLWH (unpublished data). This is consistent with the hypothesis that LPS is related to the mDC transcriptional changes that we observed in PLWH. Those 49 genes we have identified in mDCs that remain altered in PLWH with ART and have not previously been identified in biological studies in PLWH, such as AOAH, are candidates for further investigation in their role in HIV pathogenesis.

The participants in this study were pre-screened for near-normal CD4 T-cell counts and overall good health to be eligible for apheresis for the isolation of mDC. Therefore, the TN PLWH in this study were skewed toward long-term non-progressors, who represent only ~5–15% of the PLWH population (75), and therefore, this cohort does not reflect all TN PLWH. The majority of PLWH are progressors, who have a decline in CD4 T cells to ≤350 cells/μl within the first 7 years post-HIV infection, at which time ART is initiated. It is not yet known whether the mDC transcriptional changes we observed are also seen in HIV progressors before ART treatment.

One of the ART-treated PLWH in this study was not completely virally suppressed, having plasma viral RNA loads of ~400 copies/ml. As we only collected measurements at one time point for the participants, we cannot ascertain whether this is a viral blip or ongoing viremia. In the future, it would be important to look at mDC samples from HIV progressors pre- and post-ART to determine whether the residual changes that we observed in ART-treated participants are reflected in longitudinal samples from HIV progressors.

One barrier to effective ART is the persistence of CD4 T cells that harbor latent HIV proviruses. It has been shown that mDC promote latency and HIV persistence in CD4 T cells (76, 77). Therefore, mDC should be investigated as targets of latency-reversing agents (LRAs), the current drugs used to eliminate latently HIV-infected cells in ART PLWH, since the effects of these drugs on mDC have not been delineated. Of note, the cohort we studied included PLWH who had been taking ART for over 12 years and individuals HIV infected for up to 20 years. Thus, changes in mDCs were not restored to baseline by ART despite long times post HIV infection and ART initiation. In the future, new ART protocols should be developed to target these myeloid and mDC alterations. The findings described here, of transcriptional changes in myeloid cells enriched for mDCs from PLWH, including AOAH, should be further investigated for their relationship to O.I.s, correlates of HIV disease progression, and role in HIV persistence.

## Data Availability Statement

The datasets generated for this study can be found in the National Center for Biotechnology Information Gene Expression Omnibus (GEO) under accession no. GSE139559, https://www.ncbi.nlm.nih.gov/geo/query/acc.cgi?acc=GSE139559.

## Ethics Statement

The studies involving human participants were reviewed and approved by the National Institute of Allergy and Infectious Diseases or the Vaccine and Gene Therapy Institute Florida. The patients/participants provided their written informed consent to participate in this study.

## Author Contributions

SM was responsible for conceptualizing, writing the manuscript, and performing the experiments. YZ performed the experiments and contributed to the methods. DD and RS were responsible for conceptualizing the experiments as well as advising and funding of the project.

### Conflict of Interest

The authors declare that the research was conducted in the absence of any commercial or financial relationships that could be construed as a potential conflict of interest.
