# Supplementary material for: Myeloid Cells Enriched for a Dendritic Cell Population From People Living With HIV Have Altered Gene Expression Not Restored by Antiretroviral Therapy
Source: Front Immunol. 2020 Mar 4;11:261. doi: 10.3389/fimmu.2020.00261 (PMC7064632; doi:10.3389/fimmu.2020.00261)

Supplementary Figures

Supplementary Figure 1. mDC and monocyte Isolation for RNA expression studies. Myeloid dendritic cells and monocytes from treatment-naïve and ART-treated PLWH and HIV uninfected individuals were isolated by elutriation and (a) antibody-based enrichment followed by (b) fluorescence activated cell sorting.

A.

Cell Surface Marker Enrichment	mDC	monocytes
CD1c	+	-
BDCA-4	-	-
CD19	-	-

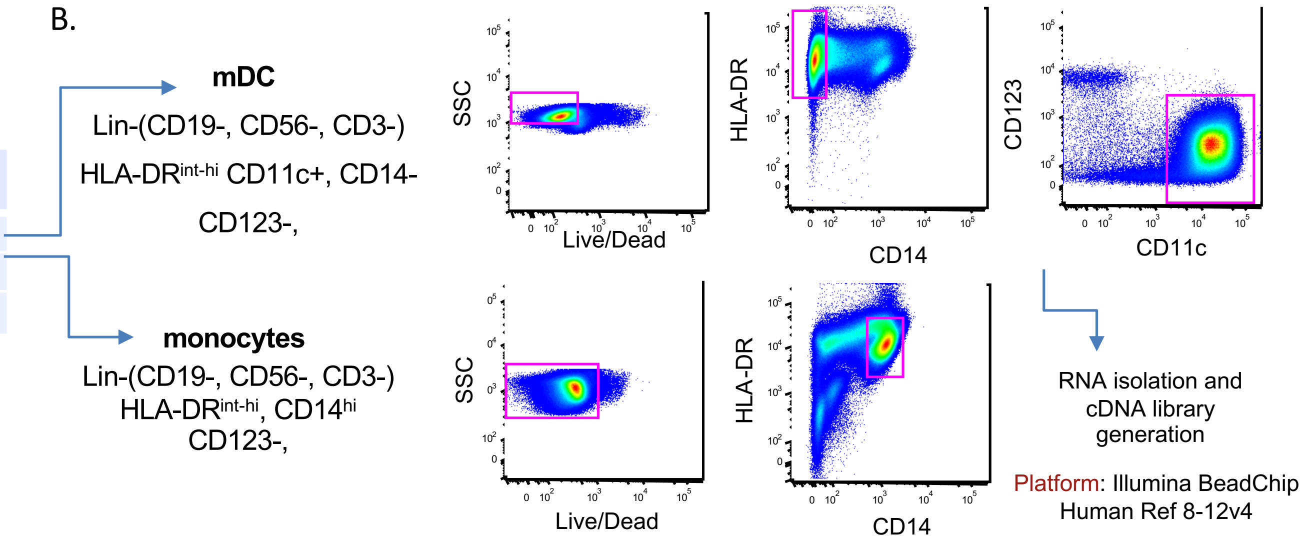

Supplementary Figure 2. Type I Interferon pathways dominate alongside suppression of the NF-κB pathway during TN HIV infections, but these pathways are restored to basal levels with ART. Apoptosis, phagocytosis, and DC- lymphoid cell crosstalk pathways remain altered even after ART. The majority of significantly altered pathways in mDC during HIV infection are altered in treatment-naïve PLWH with some pathways overlapping with or are unique to those from ART-treated PLWH.

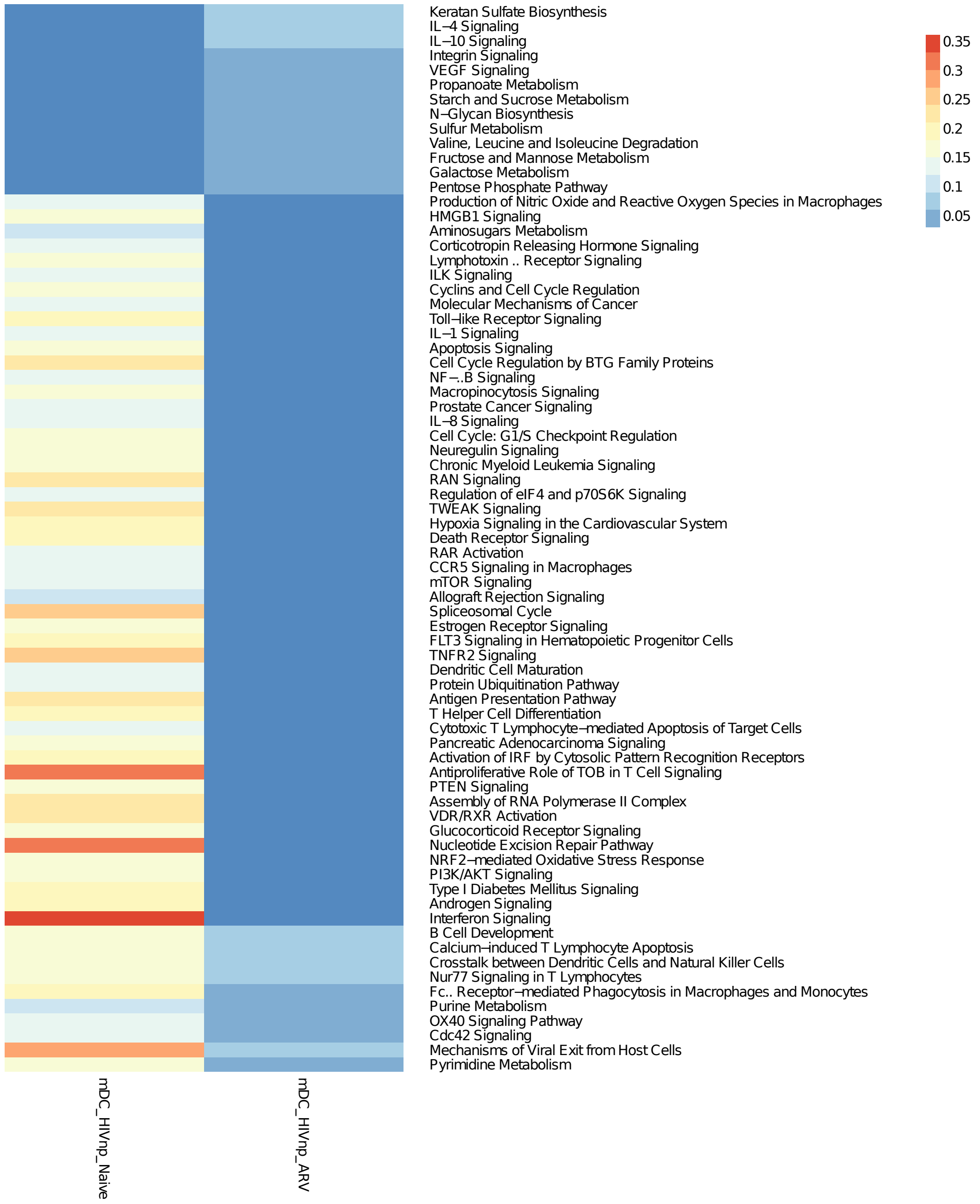

Supplementary Figure 3. mDC in PLWH irrespective of treatment status have alterations in myeloid lineage and inflammation genes compared to HIV uninfected individuals. Illustration of Gene Set Enrichment Analysis (GSEA) of selected significant modules and representative individual genes showing the maintenance of gene transcription in mDC from PLWH. This also shows the reversal of alterations in interferon pathways with ART, with IFN sets upregulated in mDC from TN PLWH and downregulated in ART-treated PLWH compared to HIV uninfected individuals.

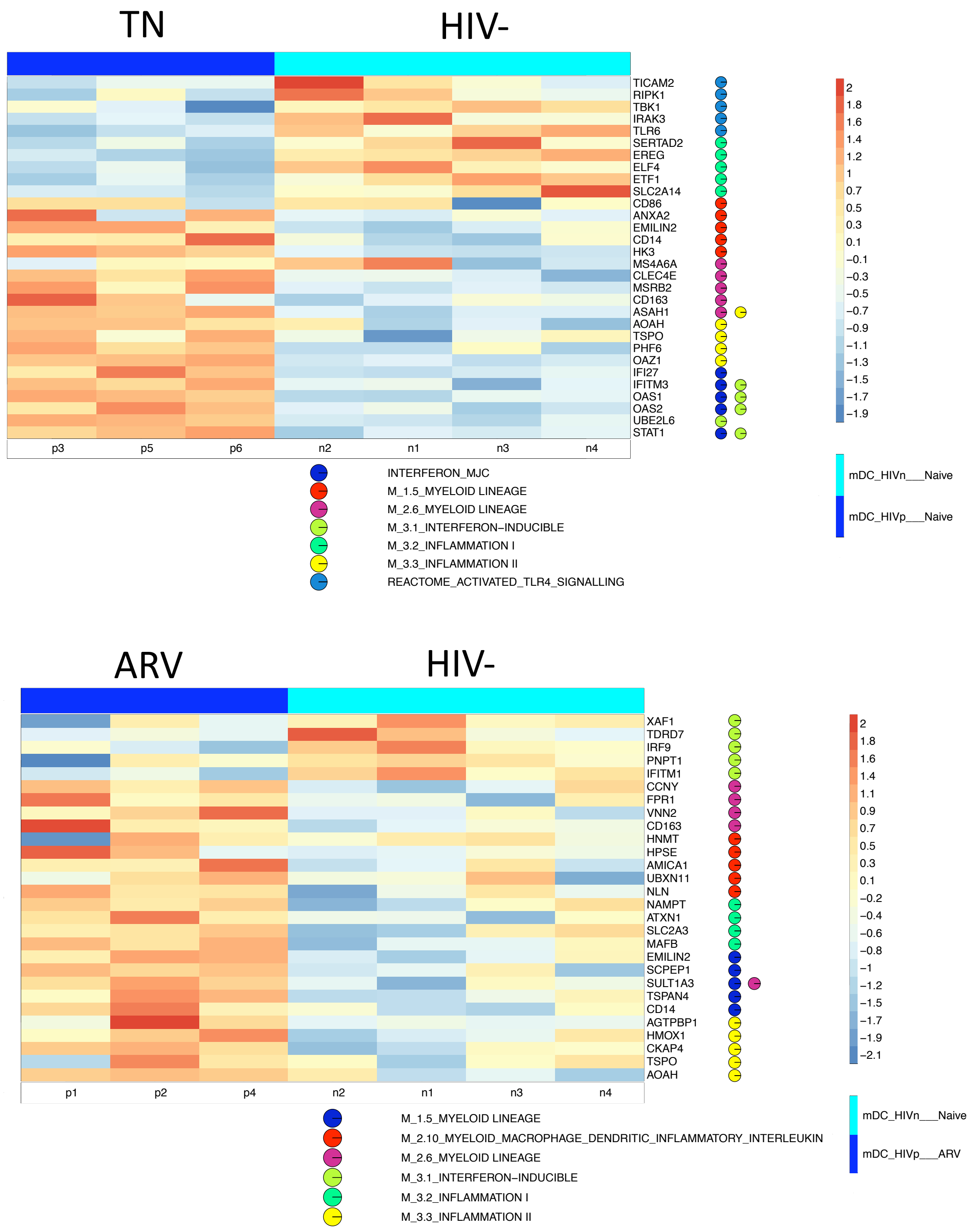

Supplement: Supplementary file 1 [file Data_Sheet_1.PDF]
